# Evaluation of the Quality and Reliability of ChatGPT‐4's Responses on Allergen Immunotherapy Using Validated Instruments for Health Information Quality Assessment

**DOI:** 10.1002/clt2.70130

**Published:** 2025-11-29

**Authors:** Ivan Cherrez‐Ojeda, Torsten Zuberbier, Gabriela Rodas‐Valero, Jorge Sanchez, Michael Rudenko, Stephanie Dramburg, Pascal Demoly, Davide Caimmi, René Maximiliano Gómez, German D. Ramon, Ghada E. Fouda, Kim R. Quimby, Herberto Chong‐Neto, Oscar Calderon Llosa, Jose Ignacio Larco, Olga Patricia Monge Ortega, Marco Faytong‐Haro, Oliver Pfaar, Jean Bousquet, Karla Robles‐Velasco

**Affiliations:** ^1^ Institute for Allergology Charité—Universitätsmedizin Berlin Corporate Member of Freie Universität Berlin and Humboldt‐Universität zu Berlin Berlin Germany; ^2^ Fraunhofer Institute for Translational Medicine and Pharmacology ITMP, Immunology and Allergology Berlin Germany; ^3^ Universidad Espiritu Santo Samborondon Ecuador; ^4^ Respiralab Research Group Guayaquil Ecuador; ^5^ Group of Clinical and Experimental Allergy Hospital “Alma Mater de Antioquia” University of Antioquia Medellín Colombia; ^6^ London Allergy and Immunology Center London UK; ^7^ Department of Pediatric Respiratory Care Immunology and Intensive Care Medicine Charité Universitätsmedizin Berlin Berlin Germany; ^8^ Department Respiratory Medicine and Allergy Allergy Unit Hôpital Arnaud de Villeneuve University Hospital of Montpellier Montpellier France; ^9^ IDESP, University Montpellier ‐ INSERM, and Department of Pulmonology, Division of Allergy University of Montpellier INSERM Montpellier France; ^10^ Faculty of Health Sciences Catholic University of Salta Salta Argentina; ^11^ Hospital Italiano Regional del Sur Bahía Blanca Argentina; ^12^ Food and Drug Allergy Center Cairo Egypt; ^13^ George Alleyne Chronic Disease Research Centre Caribbean Institute for Health Research The University of the West Indies Bridgetown Barbados; ^14^ Department of Pediatrics Division of Allergy and Immunology Complexo Hospital de Clínicas Federal University of Paraná Curitiba Brazil; ^15^ Clínica SANNA el Golf Lima Peru; ^16^ Allergy Department Clinica San Felipe Lima Peru; ^17^ Allergology Unit San Juan de Dios Hospital San José Costa Rica; ^18^ University of Costa Rica San José Costa Rica; ^19^ Facultad de Investigación Universidad Estatal de Milagro Milagro Guayas Ecuador; ^20^ Instituto de Investigación Universidad Agraria del Ecuador Guayaquil Ecuador; ^21^ Department of Otorhinolaryngology, Head and Neck Surgery, Section of Rhinology and Allergy University Hospital Marburg, Philipps‐Universität Marburg Marburg Germany

**Keywords:** allergen immunotherapy, allergic rhinitis, artificial intelligence

## Abstract

**Background:**

Chat Generative Pre‐Trained Transformer 4 (ChatGPT‐4) represents an advancing large language model (LLM) with potential applications in medical education and patient care. While Allergen Immunotherapy (AIT) can change the course of allergic diseases, it can also bring uncertainty to patients, who turn to readily available resources such as ChatGPT‐4 to address these doubts. This study aimed to use validated tools to evaluate the information provided by ChatGPT‐4 regarding AIT in terms of quality, reliability, and readability.

**Methods:**

In accordance with EAACI clinical guidelines about AIT, 24 questions were selected and introduced in ChatGPT‐4. Independent reviewers evaluated ChatGPT‐4 responses using three validated tools: the DISCERN instrument (quality), JAMA Benchmark criteria (reliability), and Flesch‐Kincaid Readability Tests (readability). Descriptive statistics summarized findings across categories.

**Results:**

ChatGPT‐4 responses were generally rated as “fair quality” on DISCERN, with strengths in classification/formulations and special populations. Notably, the tool provided good‐quality responses on the preventive effects of AIT in children and premedication to reduce adverse reactions. However, JAMA Benchmark scores consistently indicated “insufficient information” (median = 0–1), primarily due to absent authorship, attribution, disclosure, and currency. Readability analyses revealed a college graduate–level requirement, with most responses classified as “very difficult” to understand. Overall, ChatGPT‐4 demonstrated fair quality, insufficient reliability, and difficult readability for patients.

**Conclusions:**

ChatGPT‐4 provides generally well‐structured responses on AIT but lacks reliability and readability for clinical or patient‐directed use. Until specialized, reference‐based models are developed, healthcare professionals should supervise its use, particularly in sensitive areas such as dosing and safety.

## 1 Introduction

Chat Generative Pre‐Trained Transformer (ChatGPT), a state‐of‐the‐art artificial intelligence tool based on language models, has the ability to maintain a human‐like conversational dialog and has come to transform the healthcare field [1]. The latest version, ChatGPT‐4, released in March 2023, has evolved into a large multimodal model capable of processing image and text inputs and producing text outputs. In addition, it has the potential to improve the accuracy over its predecessor [2] which exhibited a propensity to generate fictional, erroneous, or unsubstantiated information in response to queries, a phenomenon referred to as “hallucination” [3]. ChatGPT has over 100 million weekly users, and despite it was not created for healthcare sector, it has potential applications in: (i) education support, more than 50% of students in health careers are using it for obtaining information [4, 5]; (ii) clinical support, such as providing assistance to doctors in diagnosis and treatment and as a patient education tool [6, 7, 8] and (iii) disciplinary development, integrating disciplines and talent cultivation [8].

Notably, ChatGPT‐4 demonstrates the highest medical domain knowledge of large language models (LLM) to date [9]. Coupled with free access, this feature enhanced the attraction of healthcare professionals who were interested in self‐education but were hindered by limited access to clinical journals and guidelines [9].

In the allergy field, primary care and family physicians are frequently the first responders in diagnosing and managing allergic symptoms including the use of Allergen Immunotherapy (AIT) [10, 11]. AIT has been a successful therapeutic option for allergic rhinitis, rhinoconjunctivitis, and allergic asthma for more than 50 years. To date, it is the only treatment that has demonstrated the ability to modify TH2‐directed immune responses and reduce allergic nasal and ocular symptoms upon chronic or recurrent exposure to aeroallergens, thereby significantly altering the course of the disease [12]. AIT not only alleviates symptoms by promoting immunological tolerance to the allergen but also can modify the course of allergic disorders [13]. This includes preventing the escalation of symptoms, the development of new sensitivities, and the onset of asthma [13]. Furthermore, among its advantages, the beneficial effects of AIT can persist even after treatment discontinuation; it is also clinically effective, cost‐effective, and disease‐modifying compared to standard drugs. Moreover, starting AIT during early childhood has been shown to significantly decrease the likelihood of developing asthma, concurrently lowering healthcare costs [14, 15].

However, despite its historical efficacy and safety, it remains underutilized [16, 17]. Potential reasons, particularly at the primary care level, include a lack of knowledge and a low level of confidence in using AIT [11], as shown by a previous survey where only 23% of participant general practitioners evidenced adequate knowledge [18]. One study reported a lack of AIT training as one of the primary reasons for not recommending AIT [18].

Given the critical nature of AIT, including its specific dosing regimens, safety protocols, and contraindications, verifying the accuracy of ChatGPT‐4 outputs about this topic is vital to avoid the propagation of misinformation that could lead to suboptimal patient outcomes. Incorporating artificial intelligence (AI) tools like ChatGPT‐4 into medical education and patient care can align technological advancements with healthcare goals [19]. However, a systematic evaluation of ChatGPT‐4's reliability, accuracy, and readability in AIT is a necessary step to ensure that it complements and enhances healthcare delivery rather than introducing new challenges. For this reason, we aim to assess the quality, reliability, and readability of ChatGPT‐4 responses to queries regarding AIT utilizing validated instruments.

## 2 Methods

### 2.1 Study Design

This was a single‐stage study designed to evaluate the medical information generated by ChatGPT‐4 (OpenAI). As a first step, a comprehensive review of the international European Academy of Allergy and Clinical Immunology (EAACI) guidelines on Allergen Immunotherapy (AIT) was undertaken [20]. These guidelines were selected given their international recognition, multidisciplinary authorship, and role as the most authoritative and up‐to‐date consensus on the indications, contraindications, efficacy, safety, and clinical use of AIT. They provided the conceptual foundation for the development of key domains and questions used to systematically explore ChatGPT‐4 outputs.

### 2.2 Question Selection and Data Collection

Based on the EAACI [20] position paper on AIT, several key domains were identified which were stratified into six categories, including (i) the definition of AIT; (ii) classification/formulations; (iii) general indications, absolute and relative contraindications; (iv) the use of standardized products with demonstrated efficacy; (v) its application in special populations such as patients with coexisting asthma, children, the elderly, pregnant women, and polysensitized individuals; and (vi) adverse events related to AIT, recommendations to ensure a safe and well‐tolerated treatment course, and risk factors for systemic reactions. These domains were transformed into questions, which served as the foundation for structured interactions with ChatGPT‐4. The 24 initial questions are presented in Table S1.

Each question was first shared individually with independent reviewers who, through a consensus process conducted in an online meeting, agreed to reformulate and refine the question set to comprehensively capture issues of relevance to both physicians and patients regarding AIT. The revised questions were iteratively developed based on reviewer feedback and investigator input.

For each question extracted, a comprehensive search was conducted in ChatGPT‐4, on a single computer using Google Chrome on July 16, 2024. Each question was introduced as an independent chat session to avoid prior context bias. The outputs were subsequently collated, anonymized, and organized into a structured Excel file, which was shared individually with the panel of evaluators for systematic assessment.

### 2.3 Expert Review and Evaluation Process

To enhance inter‐rater consistency, all evaluators underwent a structured online training process led by an experienced instructor familiar with the assessment tools used in this study (namely, the DISCERN instrument, JAMA Benchmark criteria, and Flesch–Kincaid Readability Tests). The training consisted of two comprehensive calibration sessions. During these sessions, evaluators received detailed instructions on how to apply each scoring tool consistently. Following the calibration sessions, a pre‐test was administered to assess how consistently and effectively the raters were using the tools. After completing the pre‐test, a brief online discussion was held to compare scores and clarify any discrepancies, ensuring proper alignment and calibration among the reviewers.

Once calibration was confirmed, evaluators received the final Excel spreadsheet containing all the material to be assessed. The evaluations were conducted independently to minimize the risk of bias. The evaluators were blinded to each other's scores. After a period of approximately 2 months, all ratings were collected. One investigator then compiled, cleaned, and coded the data to anonymize the results and prepare them for statistical analysis.

The tools used to assess quality, reliability, and readability, are briefly described below.

#### 2.3.1 DISCERN

The DISCERN score was developed to provide a quality assessment tool for written patient information regarding management options. It consists of 15 key questions and an overall quality rating. Each key question represents a separate quality criterion—an essential feature or standard that is important for good‐quality information on treatment choices. The tool can be separated into two categories—Q1–Q8, which assess the reliability of the information, and Q9–Q15, which covers the details of the information about treatment choices [21] (Table 1). Considering DISCERN is applied to questions regarding treatment, AIT could be evaluated by this tool across its different categories.

**TABLE 1:** Tools to assess the quality of information provided by ChatGPT‐4.

Name	Components	Scoring
DISCERN [21, 22]	Q1–Q8: Reliability.	Maximum score: 80 points.
		Excellent quality: +63.
	Q9–Q15: Details of the information about treatment choices.	Good quality: 51 to 62.
		Fair: 39 to 50.
	Q16: Overall quality rating.	Poor: 27 to 38.
		Very poor: 16 to 26.
JAMA benchmark [23]	Authorship	0 to 1.9 point: Insufficient information.
	Attribution	2.0 to 3.9 points: Partially sufficient information.
	Disclosure	4 points: Completely sufficient information.
	Currency	
Flesch‐Kincaid reading ease score [24]	Flesch reading ease score = 206.835 − 1.015 × (Total words/Total sentences) − 84.6 × (Total syllables/Total words).	Flesch reading ease score
		Very difficult: ≤ 29.
		Difficult: 30–49.
		Fairly difficult: 50–59.
		Standard: 60–69.
		Fairly easy: 70–79.
		Easy: 80–89.
		Very easy: ≥ 90.
Flesch–Kincaid grade level	Flesch–Kincaid grade level = 0.39 × (Total words/Total sentences) + 11.8 × (Total syllables/Total words) − 15.59.	Grade 1–2: 1.0–2.9.
		Grade 3–4: 3.0–4.9.
		Grade 5–6: 5.0–6.9.
		Grade 7–8: 7.0–8.9.
		Grade 9–10.9: 9.0–10.9.
		Grade 11–12 (high school): 11.0–12.9.
		College (undergraduate): 13.0–15.9.
		College graduate/Professional: 16.0+.

#### 2.3.2 JAMA Benchmark

The JAMA benchmark criteria were developed to assess the reliability and quality of online information, particularly in the context of patient educational and medical transparency. It comprises four standards: authorship, attribution, disclosure, and currency, with each one scoring 1 point [23] (Table 1). An overall score of 0–1 point represents insufficient information, 2 to 3 points represents partially sufficient information, and 4 points represents completely sufficient information [25].

### 2.4 Readability Analysis

Readability of a written text is an objective measure of the reading skills an individual must possess to understand that material, and it is evaluated in terms of “grade levels”. The Flesch Reading Ease Score (FRES) score is the earliest of the commonly used tools to assess readability, and it is followed by The Flesch‐Kincaid Grade Level (FKGL), a modified version of the previous scale and the most widely used tool to assess readability [24, 26, 27].

These two scales use word and sentence length in formulas to provide a score of readability and education level, by the United States (US) grade level, of a piece of text [27] (Table 2). To calculate the scores, we used an online calculator for FRES and FKGL, which can be found at https://goodcalculators.com/flesch‐kincaid‐calculator/.

**TABLE 2:** Flesch‐Kincaid reading ease score categories.

Flesch reading ease score	Readability level/Category	Estimated reading grade
0–29	Very difficult	College graduate/Professional
30–49	Difficult	13th to 16th grade (college)
50–59	Fairly difficult	10th to 12th grade
60–69	Standard	8th or 9th grader
70–79	Fairly easy	7th grader
80–89	Easy	6th grader
90–100	Very easy	5th grader

### 2.5 Statistical Methods

Descriptive statistics were calculated according to the distribution of each variable: for parametric distributions, results are presented as mean ± standard deviation; for non‐parametric distributions, results are presented as median and interquartile range (IQR). The scores for DISCERN, JAMA, FKGL, and FRES were calculated according to the cutoffs described in Table 1.

### 2.6 Role of the Funding Source

This research received no external funding.

## 3 Results

**FIGURE 1:**
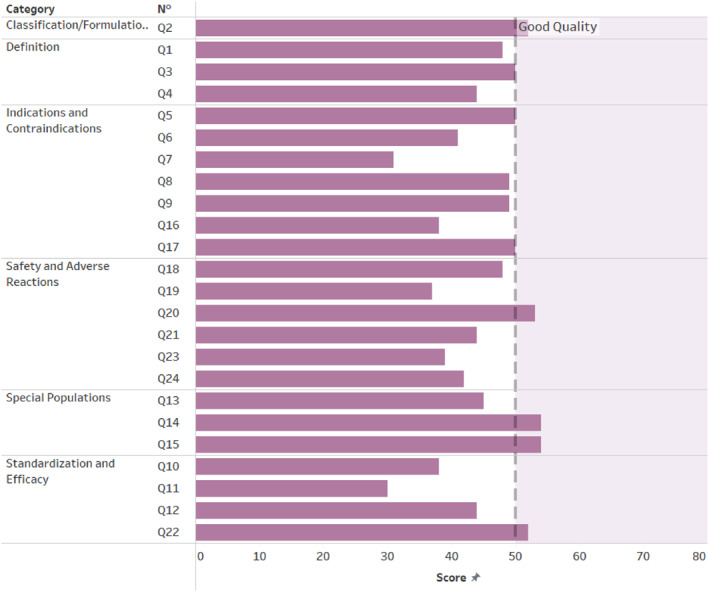
Quality scores according to DISCERN.

Figure 1 and Table 3 present a comprehensive analysis of the median scores given by reviewers to responses of ChatGPT‐4 across six categories: Definition, Classification/Formulations, Indications and Contraindications, Standardization and Efficacy, Special Populations, and Safety and Adverse Reactions. The overall assessment was based on these categories.

**TABLE 3:** Evaluation of ChatGPT‐Generated Answers on Allergen Immunotherapy: Quality, Reliability, and Readability Scores with Interpretation.

Category	Question	DISCERN		JAMA benchmark		FRES/FKGL	
		Score	Interpretation	Score	Interpretation	Score	Interpretation
Definition	Q1: What is allergen immunotherapy (AIT)?	48	Fair quality	0	Insufficient information	32.9	Difficult to read (college)
	Q3: What is subcutaneous immunotherapy (SCIT)	50	Fair quality	1	Insufficient information	41.1	Difficult to read (college)
	Q4: What is sublingual immunotherapy (SLIT)?	44	Fair quality	0	Insufficient information	34.3	Difficult to read (college)
	Median (IQR)	48 (6)	Fair quality	0 (1)	Insufficient information	34.3 (8.2)	Difficult to read (college)
Classification/Formulations	Q2: Which are the available formulations for allergen immunotherapy?	52	Good quality	1	Insufficient information	32.2	Difficult to read (college)
	Median (IQR)	52 (−)	Good quality	1 (−)	Insufficient information	32.2 (−)	Difficult to read (college)
Indications and contraindications	Q5: Which are the general indications for using allergen immunotherapy?	50	Fair quality	0	Insufficient information	24	Very difficult to read (college graduate)
	Q6: What are the contraindications of allergen immunotherapy?	41	Fair quality	0	Insufficient information	8.5	Very difficult to read (college graduate)
	Q7: In what patients is it recommended to use sublingual immunotherapy (SLIT)?	31	Poor quality	0	Insufficient information	46.4	Difficult to read (college)
	Q8: What type of immunotherapy and duration of treatment is recommended for grass pollen‐driven allergic rhinitis in adults?	49	Fair quality	0	Insufficient information	33.4	Difficult to read (college)
	Q9: What type of immunotherapy and duration of treatment is recommended for perennial house dust mite allergic rhinitis in adults?	49	Fair quality	1	Insufficient information	29.3	Very difficult to read (college graduate)
	Q16: What is the recommended duration of allergen immunotherapy in order to achieve long‐term efficacy after discontinuation in patients with allergic rhinitis?	38	Poor quality	1	Insufficient information	22.5	Very difficult to read (college graduate)
	Q17: Is controlled asthma a contraindication to receiving allergen immunotherapy?	50	Fair quality	1	Insufficient information	12.6	Very difficult to read (college graduate)
	Median (IQR)	49 (12)	Fair quality	0 (1)	Insufficient information	24 (20.8)	Very difficult to read (college graduate)
Standardization and efficacy	Q10: What is the importance of using standardized allergen products for allergen immunotherapy?	38	Poor quality	0	Insufficient information	15.8	Very difficult to read (college graduate)
	Q11: Which are effective pollen mixtures frequently used for allergen immunotherapy?	30	Poor quality	0	Insufficient information	39.3	Difficult to read (college)
	Q12: Does the efficacy of allergen immunotherapy change if a patient with allergic rhinitis has also co‐existing asthma?	44	Fair quality	0	Insufficient information	25.6	Very difficult to read (college graduate)
	Q22: What are the preventive effects of allergen immunotherapy for children and adolescents with allergic rhinitis driven by pollen allergy?	52	Good quality	1	Insufficient information	15.6	Very difficult to read (college graduate)
	Median (IQR)	41 (18)	Fair quality	0 (1)	Insufficient information	20.7 (20.2)	Very difficult to read (college graduate)
Special populations	Q13: Can allergen immunotherapy be given to pediatric patients with allergic rhinitis?	45	Fair quality	1	Insufficient information	11.8	Very difficult to read (college graduate)
	Q14: Is the use of allergen immunotherapy recommended in otherwise healthy elderly adults with allergic rhinitis?	54	Good quality	2	Partially sufficient information	12.6	Very difficult to read (college graduate)
	Q15: Is it recommended to initiate allergen immunotherapy regimen for allergic rhinitis during pregnancy?	54	Good quality	1	Insufficient information	23.6	Very difficult to read (college graduate)
	Median (IQR)	54 (9)	Good quality	1 (1)	Insufficient information	12.6 (11.8)	Very difficult to read (college graduate)
Safety and adverse reactions	Q18: What are the most common adverse reactions to subcutaneous immunotherapy?	48	Fair quality	1	Insufficient information	29.2	Very difficult to read (college graduate)
	Q19: At what time can most adverse reactions occur after the application of subcutaneous immunotherapy?	37	Poor quality	1	Insufficient information	34.3	Difficult to read (college)
	Q20: Which premedication can be used to reduce the severity of local and systemic cutaneous reactions to allergen immunotherapy?	53	Good quality	1	Insufficient information	0	Very difficult to read (college graduate)
	Q21: Should premedication with an oral H1 antihistamine be recommended before allergen immunotherapy?	44	Fair quality	1	Insufficient information	8.2	Very difficult to read (college graduate)
	Q23: Which are risk factors for the appearance of systemic reactions during allergen immunotherapy?	39	Fair quality	1	Insufficient information	25.7	Very difficult to read (college graduate)
	Q24: Is allergen immunotherapy considered safe and well tolerated in patients?	42	Fair quality	0	Insufficient information	33	Difficult to read (college)
	Median (IQR)	43 (10.8)	Fair quality	1 (0)	Insufficient information	27.5 (27.2)	Very difficult to read (college graduate)
Overall score	Median (IQR)	46.5 (10.5)	Fair quality	1 (1)	Insufficient information	25.7 (20)	Very difficult to read (college graduate)

*Note:* Green indicates good quality, meaning the information is complete, clear, and easily interpretable. Yellow represents fair quality or moderate difficulty, signaling sections that are understandable but may contain minor gaps or elements that are less straightforward. Red denotes poor quality or insufficient information, identifying areas where the content was difficult to read, incomplete, or required substantial clarification.

### 3.1 Definition

This category included three questions. The DISCERN median scores indicated that most replies were of “fair quality”. JAMA Benchmark showed that ChatGPT‐4 replies provided “insufficient information” with a median score = 1. In terms of readability, the text was difficult to understand, and according to FKGL, a college graduate level was needed to understand it.

### 3.2 Classification/Formulations

The DISCERN score for this question was classified as “good quality.” However, the JAMA median score showed that ChatGPT‐4 response provided “insufficient information” (median score 1). The FKGL score indicated that a college level of proficiency was required to understand the text and was difficult to understand.

### 3.3 Indications and Contraindications

This section included seven questions. Within this area, the DISCERN median scores indicated that most replies fell into the “fair quality” category, with only two questions having responses classified as “poor quality”. The median JAMA score for this section was 0, indicating “insufficient information.” In this section, the FKGL scores suggest a need for college graduate‐level proficiency to understand the text.

### 3.4 Standardization and Efficacy

Questions addressing the importance of using standardized allergen products for AIT and the effectiveness of pollen mixtures in AIT obtained the lowest scores, reflecting responses of poor quality. In contrast, questions regarding the efficacy of AIT in patients with allergic rhinitis (AR) and asthma, as well as the preventive effects of AIT in children and adolescents with pollen allergy, were rated as fair and good quality, respectively. Nevertheless, the median JAMA score for this section was 0, indicating “insufficient information.” Furthermore, the readability analysis showed that college graduate‐level proficiency was required to fully comprehend the text.

### 3.5 Special Populations

We included three questions in this area. DISCERN median scores indicated that most replies fell into the “good quality” category, with only one response classified as “fair quality”. The JAMA median ratings for all the questions indicated “insufficient information”. The FKGL results were consistent with previous classifications, requiring a college graduate‐level education to understand.

### 3.6 Safety and Adverse Reactions

This section comprised six questions. Based on DISCERN scores, most responses were rated as “fair quality,” with only one, concerning pre‐medication to reduce the severity of reactions to AIT, classified as “good quality.” In contrast, the question about the time of onset of adverse reactions after AIT received the lowest quality rating. The median JAMA scores for all questions indicated “insufficient information.” Readability analysis using FKGL was consistent with these findings, showing that a college graduate‐level education was required for comprehension.

### 3.7 Overall Assessment

When evaluating the overall performance across all categories, the DISCERN median scores indicated a fair quality of the information provided. The overall JAMA median score suggested insufficient information in the answers (median score = 1). Additionally, the overall FKGL and FRES scores revealed that the information provided by ChatGPT‐4 required a “college graduate” level to comprehend and was “very difficult” to read.

## 4 Discussion

In this study, ChatGPT‐4 generally provided responses of fair quality, though the reliability of information was largely insufficient according to the JAMA Benchmark assessment across most allergen immunotherapy (AIT) categories. The generated text was written at a “college graduate” comprehension level, frequently rated as “very difficult” to read. Despite these limitations, ChatGPT‐4 demonstrated notable strengths, particularly within the “Classification/Formulations” and “Special Populations” categories, as well as for specific queries such as the preventive effects of AIT in children and adolescents with pollen‐induced allergic rhinitis and the use of premedication to reduce adverse reactions during AIT (Table 4). These strengths likely reflect the limited number of AIT formulations (subcutaneous and sublingual immunotherapy) [20]; and the consistent representation of these treatments in guidelines and educational resources, making them more readily identifiable and reproducible for this tool.

**TABLE 4:** Queries with good quality answers.

Question	Category
Which are the available formulations for allergen immunotherapy?	Classification/Formulations
What are the preventive effects of allergen immunotherapy for children and adolescents with allergic rhinitis driven by pollen allergy?	Standardization and efficacy
Is the use of allergen immunotherapy recommended in otherwise healthy elderly adults with allergic rhinitis?	Special populations
Is it recommended to initiate allergen immunotherapy regimen for allergic rhinitis during pregnancy?	Special populations
Which premedication can be used to reduce the severity of local and systemic cutaneous reactions to allergen immunotherapy?	Safety and adverse reactions

However, ChatGPT‐4 failed to meet the JAMA Benchmark criteria for reliability, which assess four specific attributes (authorship, attribution, disclosure, and currency), across all categories, thereby limiting the trustworthiness of its responses. Omitting even two of these components results in a categorization of incomplete or insufficient reliability. Given the small number of criteria, responses can easily score zero, even when the medical content itself is clinically accurate. For example, when prompted for bibliographic references, ChatGPT‐4 frequently cited general sources such as AAAAI, EAACI, or guideline and journal titles but did not provide specific authors, years, or detailed citations. In question Q20, concerning premedication to reduce AIT‐related adverse reactions, the response aligned with guideline‐based clinical practice; nonetheless, the absence of structured references led to a JAMA score of “insufficient,” despite a DISCERN score of 53, indicating good quality. Similarly, in question Q12, regarding the efficacy of AIT in patients with co‐existing asthma, the content was accurate but lacked precise citations, resulting in a JAMA score of zero while DISCERN rated the response as fair to good.

These findings highlight the conceptual difference between DISCERN and JAMA. The DISCERN instrument evaluates quality using a multidimensional framework, considering clarity of aims, relevance, source usage, balance, acknowledgment of uncertainty, and the quality of information on treatment options, including benefits, risks, and implications for shared decision‐making [21]. In contrast, JAMA adopts a narrower, structural approach requiring explicit authorship, attribution, disclosure, and currency [23, 28]. Consequently, it is common for ChatGPT‐4 responses to be rated as fair or good by DISCERN for content clarity and balance, yet fail under JAMA due to the absence of formal references. This distinction highlights the importance of using complementary assessment tools: DISCERN assesses substantive quality, whereas JAMA evaluates formal reliability. Also, the complexity of the generated text suggests that ChatGPT‐4 is better suited for physician education than for patient‐directed materials. While the tool provides generally accurate information in some areas, its accessibility for patients remains limited. Emerging tools such as Consensus GPT, which provides direct access to over 200 million academic papers, may offer more reliable, clinically useful information for professional users [29].

Previous studies have similarly evaluated ChatGPT models in patient education across medical specialties. Kasapovic et al. [30] found that ChatGPT‐3.5 provided moderate to low‐quality information regarding orthopedic and trauma procedures, whereas Brock et al. [31] demonstrated higher‐quality but less readable content for informed consent in carpal tunnel surgery. Riestra‐Ayora et al. [32] confirmed ChatGPT‐3.5's accuracy in responses about prevalent rhinology pathologies, and Burnette et al. [33] found ChatGPT‐4 delivered generally accurate and complete information on immune‐related adverse events. However, these studies often lacked the use of validated quality metrics, limiting their generalizability.

Despite improvements in reliability from ChatGPT‐3.5 to ChatGPT‐4, hallucinations remain a concern, with rates of 28.6% versus 39.6%, respectively [34]. LLMs also inherit the limitations of their underlying data, which include variability in guideline quality and perspectives. For instance, certain aspects addressed by Q7 (patients recommended for sublingual immunotherapy, SLIT), Q16 (recommended duration of AIT for long‐term efficacy), Q10 (importance of using standardized allergen products), and Q11 (effective pollen mixtures frequently used for AIT) posed challenges for ChatGPT‐4 to address with good quality and full reliability. While these topics are covered in guidelines, nuances such as identifying optimal patient populations for SLIT, specifying the duration necessary for sustained efficacy, or detailing appropriate standardized allergen products and pollen mixtures are less consistently presented across publicly available resources. This variability might increase the likelihood of incomplete or inaccurate responses. In line with this, an EAACI review of AIT guidelines published between 1980 and 2016 identified that among 31 guidelines, many of them scored poorly on Appraisal of Guidelines Research and Evaluation (AGREE II) assessment tool, particularly regarding applicability, rigor, and stakeholder involvement [35], in consequence, high‐quality LLM outputs, will therefore, depend on reliable, representative source data that reflects diverse clinical contexts, including low‐income settings.

### 4.1 Strengths and Limitations

This study's key strength lies in its innovative focus on LLM evaluation in a complex and clinically specialized domain. The use of validated assessment tools ensures methodological rigor, enhancing the credibility of our findings. Structured scoring provides an objective framework for comparing LLM‐generated responses with clinical standards. Limitations include the reliance on pre‐defined guideline‐based questions, which may not fully capture the breadth of inquiries encountered in clinical or patient settings, and the fact that inter‐rater agreement among the seven allergists was not formally assessed. Although all evaluations were conducted independently by seven experienced allergists following structured training and calibration sessions, formal statistical assessment of inter‐rater agreement was not performed. This was a pragmatic decision due to the consensus‐based refinement process of the questions and the study's focus on exploratory evaluation of LLM outputs.

### 4.2 Implications and Future Directions

Our findings underscore that while ChatGPT‐4 shows promise, it cannot yet replace professional judgment or serve as a standalone, reliable patient education tool in AIT. The development of LLM systems tailored for allergy practice, coupled with collaboration between clinicians and LLM engineers, is essential to improve accuracy, readability, and reliability. Future models must integrate structured references, ensure contextual understanding, and account for the quality and diversity of training data to mitigate bias and enhance clinical applicability [36].

## 5 Conclusion

ChatGPT‐4 provides fair‐quality responses on allergen immunotherapy (AIT) and correctly reflects its efficacy in allergic rhinitis, asthma, and atopic dermatitis. However, outputs often lack references, precision, and readability, limiting their reliability for patients. Healthcare professionals should supervise ChatGPT's use for sensitive topics such as dosing, treatment selection, and safety. Future work should focus on refining AI tools to enhance accuracy, clinical relevance, and patient accessibility, allowing them to reliably complement evidence‐based care.

## Author Contributions

**Ivan Cherrez‐Ojeda:** conceptualization, investigation, writing – original draft, funding acquisition, methodology, writing – review and editing, supervision, resources, project administration, visualization. **Torsten Zuberbier:** conceptualization, investigation, writing – original draft, writing – review and editing, formal analysis. **Gabriela Rodas‐Valero:** conceptualization, investigation, writing – original draft, writing – review and editing, formal analysis, supervision, visualization, project administration. **Jorge Sanchez:** conceptualization, investigation, writing – original draft, writing – review and editing. **Michael Rudenko:** conceptualization, investigation, writing – original draft, writing – review and editing. **Stephanie Dramburg:** conceptualization, writing – review and editing, writing – original draft, investigation. **Pascal Demoly:** conceptualization, writing – original draft, writing – review and editing, investigation. **Davide Caimmi:** conceptualization, investigation, writing – review and editing, writing – original draft. **René Maximiliano Gómez:** conceptualization, funding acquisition, writing – original draft, writing – review and editing. **German D. Ramon:** conceptualization, writing – original draft, writing – review and editing, validation. **Ghada E. Fouda:** conceptualization, writing – review and editing, writing – original draft. **Kim R. Quimby:** writing – original draft, writing – review and editing. **Herberto Chong‐Neto:** writing – original draft, writing – review and editing. **Oscar Calderon Llosa:** writing – original draft, conceptualization, writing – review and editing, investigation, visualization. **Jose Ignacio Larco:** writing – original draft, writing – review and editing. **Olga Patricia Monge Ortega:** writing – original draft, writing – review and editing. **Marco Faytong‐Haro:** writing – review and editing, writing – original draft, formal analysis, software, methodology, data curation. **Oliver Pfaar:** conceptualization, writing – original draft, writing – review and editing. **Jean Bousquet:** writing – original draft, writing – review and editing. **Karla Robles‐Velasco:** writing – original draft, writing – review and editing, supervision, resources, data curation, project administration, conceptualization, investigation, methodology, funding acquisition.

## Funding

The authors have nothing to report.

## Conflicts of Interest

D.C. reports receipt of fees for presentations at meetings from A.L.K., Stallergenes Greer, Dr. Falk, Sanofi; fees for participation in advisory boards from A.L.K., Stallergenes Greer, Puressentiel, Sanofi and AstraZeneca; and research grants (to his institution) from A.L.K. and AstraZeneca. P.D. reports fees directed to research and teaching purposes from ALK‐Abelló, AstraZeneca, Ménarini, GlaxoSmithKline, Stallergenes Greer, ThermoFisherScientific, Viatris, Zambon and consulting fees from Chiesi and Puressentiel. I.C.H.O., G.R.V., K.R.V., J.S., M.R., R.M.M., G.D.R., G.E.F., S.D., K.R.Q., H.C.N., O.C., J.I.L., O.P.M.O., M.F.H., T.Z., J.B. and O.F. declare no conflicts of interest.

## Supporting information


**Table S1**: Input questions and output responses from ChatGPT‐4.0 Regarding Allergen Immunotherapy (AIT).

## Data Availability

Data supporting the findings of this study are available from the corresponding author upon reasonable request.
