# Supplementary material for: Evaluation of the Quality and Reliability of ChatGPT‐4's Responses on Allergen Immunotherapy Using Validated Instruments for Health Information Quality Assessment
Source: Clin Transl Allergy. 2025 Nov 29;15(12):e70130. doi: 10.1002/clt2.70130 (PMC12665041; doi:10.1002/clt2.70130)
Supplement: Supplementary file 1 — Table S1: Input questions and output responses from ChatGPT‐4.0 Regarding Allergen Immunotherapy (AIT). [file CLT2-15-e70130-s001.docx]

**Supplementary Table S1.** Input Questions and Output Responses from ChatGPT-4.0 Regarding Allergen Immunotherapy (AIT)

| **Category** | **Input Questions** | **Output responses from ChatGPT-4** | **References provided by ChatGPT-4** |
| --- | --- | --- | --- |
| Definition | What is allergen immunotherapy (AIT)? | Allergen Immunotherapy (AIT), also known as allergy shots, is a long-term treatment that decreases symptoms for many people with allergic rhinitis, allergic asthma, conjunctivitis (eye allergy), or stinging insect allergy. It's a form of treatment aimed at decreasing your sensitivity to substances called allergens. Here's how it generally works:  1. **Identification of Allergens**: The treatment begins with identifying the specific allergens causing your allergic reactions through skin or blood tests.  2. **Customized Allergen Extracts**: Based on the test results, a health care provider prepares a customized allergen extract. This extract contains the allergens that trigger your allergic reactions.  3. **Gradual Exposure**: Treatment involves regular injections of the allergen extract. The initial phase involves receiving injections with increasing amounts of the allergens one to two times per week. The aim here is to gradually increase the immune system's tolerance to the allergens.  4. **Maintenance Phase**: After the effective dose is reached, the maintenance phase begins. This phase involves a consistent, effective dose of allergen, with longer periods between injections, typically about every two to four weeks.  5. **Long-term Benefits**: Over time, AIT can reduce the severity of your allergic symptoms and the need for medications to control them. It's a long-term commitment, often continuing for three to five years.  AIT is especially effective for people with allergic rhinitis, allergic asthma, and reactions to insect stings. The success rate varies among individuals and depends on the severity and type of allergy. It's important to note that AIT should only be carried out under the guidance of a healthcare professional specialized in allergies, as there are risks of allergic reactions, including anaphylaxis, associated with the treatment. | Certainly! Here are some references for further reading on Allergen Immunotherapy (AIT):  1. **American College of Allergy, Asthma & Immunology (ACAAI)**: This organization provides comprehensive information about various allergic conditions, including detailed sections on allergen immunotherapy. They offer insights into how AIT works, its effectiveness, and who might benefit from it.  2. **Asthma and Allergy Foundation of America (AAFA)**: AAFA provides educational resources about allergies and asthma. Their materials on AIT can give you a patient-centric perspective on how this treatment is used in managing allergic diseases.  3. **National Institute of Allergy and Infectious Diseases (NIAID)**: NIAID, part of the National Institutes of Health (NIH), conducts and supports research on allergic diseases. They provide scientifically vetted information on various treatments, including AIT, and their website often contains links to the latest research and clinical trials.  4. **Journal of Allergy and Clinical Immunology**: This peer-reviewed journal frequently publishes articles and studies related to allergen immunotherapy, offering a more technical and detailed perspective on current research and developments in this field.  5. **World Allergy Organization (WAO)**: The WAO is an international alliance of allergy and clinical immunology societies. They provide guidelines, position papers, and educational resources that can offer a global perspective on AIT.  6. **PubMed Central**: For those interested in more in-depth, scientific research articles, PubMed Central is a free archive of biomedical and life sciences journal literature. Searching for "allergen immunotherapy" on this platform can yield a wide range of research papers and clinical study results.  These sources are reliable for obtaining detailed and accurate information about allergen immunotherapy. |
| Classification/Formulations | Which are the available formulations for allergen immunotherapy? | Allergen immunotherapy, often referred to as allergy shots, is a long-term treatment that decreases symptoms for many people with allergic rhinitis, allergic asthma, conjunctivitis (eye allergy), or stinging insect allergy. The available formulations for allergen immunotherapy are as follows:  1. **Subcutaneous Immunotherapy (SCIT):** This is the most traditional form, where allergens are injected under the skin typically once a week for several months, then monthly for a period of 3-5 years.  2. **Sublingual Immunotherapy (SLIT):** This involves placing a tablet containing the allergen under the tongue daily. This method is used mainly for environmental allergies and has been widely used for grass and ragweed allergies.   3. **Sublingual Drops:** Similar to SLIT tablets but in a liquid form, these drops are placed under the tongue. This method is not FDA-approved in the United States but is used in some clinics.  4. **Oral Immunotherapy (OIT):** Mainly used for food allergies, this method involves consuming small, increasing amounts of the allergen in a controlled setting.  5. **Epicutaneous Immunotherapy (EPIT):** This is a newer method where allergens are delivered through a skin patch.  6. **Intralymphatic Immunotherapy (ILIT):** This is a more recent approach where allergens are injected directly into lymph nodes to achieve a quicker desensitization.  Each formulation has different indications, efficacy, side effects, and convenience factors, which are considered when choosing the best option for an individual patient. The choice depends on various factors, including the type of allergy, the age of the patient, and the patient's preference and lifestyle. | Sure, here are some references where you can find more information about the different formulations of allergen immunotherapy:  1. **American Academy of Allergy, Asthma & Immunology (AAAAI):** This professional organization provides comprehensive information on allergy treatments, including various forms of immunotherapy. They offer patient-focused resources explaining the process, effectiveness, and considerations for each type of immunotherapy.  2. **World Allergy Organization (WAO):** The WAO offers detailed information on global perspectives and practices in allergen immunotherapy. They provide guidelines, research updates, and educational resources that cover the latest developments in the field, including emerging therapies.  3. **Journal of Allergy and Clinical Immunology:** This peer-reviewed journal publishes extensive research articles, reviews, and clinical guidelines on allergy and immunotherapy. It's a great source for in-depth scientific information and the latest findings in allergen immunotherapy treatments.  4. **National Institute of Allergy and Infectious Diseases (NIAID):** NIAID provides resources on allergy research and treatments. They have patient-oriented information that explains different types of allergen immunotherapy, their benefits, risks, and ongoing research in the field.  5. **PubMed:** For more scientific and medical research papers, PubMed is a comprehensive database of medical literature. You can find numerous studies and clinical trial results related to various forms of allergen immunotherapy here.  These resources are valuable for both patients and healthcare professionals seeking detailed information about allergen immunotherapy. |
| Definition | What is subcutaneous immunotherapy (SCIT) | Subcutaneous Immunotherapy (SCIT), also known as allergy shots, is a form of treatment used for certain allergies. This therapy involves administering small, gradually increasing amounts of the allergen to the patient over time. The process works on the principle of desensitization, where the immune system becomes less sensitive to the allergen, reducing the severity of allergic reactions or preventing them altogether.  Here's a more detailed look at the process:  1. **Testing and Customization**: Initially, an allergist will test the patient to determine the specific allergens causing reactions. Based on these results, a custom-mixed vial of allergens is prepared for the patient.  2. **Initial Phase**: During the initial phase, injections of the allergen extract are given one to two times a week for several months. The dose is increased gradually to allow the immune system to build up tolerance.  3. **Maintenance Phase**: After the effective dose is reached, the treatment enters the maintenance phase. During this phase, the frequency of injections reduces, typically to once every two to four weeks. This phase can last for several years.  4. **Effectiveness**: Over time, patients may notice a decrease in symptoms when exposed to the allergen. The effectiveness of SCIT varies among individuals and depends on the severity of the allergies and the duration of treatment.  5. **Long-Term Benefits**: SCIT has been shown to provide long-term benefits in reducing allergy symptoms and can even prevent the development of new allergies and asthma in children.  It's important to note that SCIT should be administered under the supervision of a trained allergist due to the risk of allergic reactions, including anaphylaxis. Regular follow-up and adherence to the treatment schedule are essential for safety and effectiveness. | The information about Subcutaneous Immunotherapy (SCIT) was gathered from the following sources:  1. PubMed: A collection of articles and reviews on Subcutaneous Immunotherapy, discussing various aspects like effectiveness, cost, and comparison with other forms of immunotherapy【8†source】.  2. Another set of articles from PubMed, focusing on the safety aspects of SCIT, guidelines for allergen-specific immunotherapy, and a comprehensive review of systemic allergic reactions and anaphylaxis associated with allergen immunotherapy【9†source】.  3. The American College of Allergy, Asthma & Immunology (ACAAI) Public Website: This source provided detailed information on the difference between SCIT and SLIT (Sublingual Immunotherapy), the risks of allergy shots, and their effectiveness in treating allergic asthma【10†source】.  These references offer a comprehensive overview of SCIT, including its methodology, benefits, risks, and comparison with other treatments for allergies. |
| Definition | What is sublingual immunotherapy (SLIT)? | Sublingual immunotherapy (SLIT) is a treatment strategy for allergic conditions which involves placing a specially prepared allergen extract under the tongue. This method aims to increase tolerance to the allergen, thereby reducing symptoms. Here's how SLIT typically works:  1. **Allergen Identification**: First, an allergist identifies the specific allergens causing the patient's allergic reactions.  2. **Preparation of Allergen Extract**: A customized allergen extract is prepared. This can be in liquid or tablet form and contains small amounts of the identified allergen(s).  3. **Sublingual Administration**: The patient administers the allergen extract under their tongue. This is usually done daily and involves holding the extract under the tongue for a period before swallowing it.  4. **Gradual Dose Increase**: The dosage of the allergen extract is gradually increased over time. This process is designed to help the immune system become more tolerant of the allergen, thereby reducing allergic reactions.  5. **Long-term Treatment**: SLIT is a long-term treatment, often lasting several years, as it takes time to build up and maintain tolerance to allergens.  SLIT is commonly used for allergies such as allergic rhinitis and asthma triggered by pollen, dust mites, and pet dander. It's also being explored as a treatment option for food allergies. The advantages of SLIT include its convenience (it can be done at home) and non-invasive nature. However, it's crucial to undertake this treatment under medical supervision to manage potential allergic reactions. | The information about Sublingual Immunotherapy (SLIT) provided in my response is based on general knowledge and is not sourced from specific references. It's a summary of widely accepted practices and concepts in the field of allergy treatment, as understood up to my last training cut-off in April 2023. For the most current and detailed information, consulting recent medical literature or a healthcare professional specializing in allergies and immunotherapy would be advisable. |
| Indications and Contraindications | Which are the general indications for using allergen immunotherapy? | Allergen immunotherapy, also known as allergy shots, is a form of long-term treatment that decreases symptoms for many people with allergic rhinitis, allergic asthma, conjunctivitis (eye allergy), or stinging insect allergy. It's particularly considered in the following situations:  1. **Ineffectiveness of Standard Treatments:** If symptoms are not adequately controlled by standard medications like antihistamines or nasal corticosteroids, or if these medications cause unacceptable side effects.  2. **Long-Term Relief Desire:** Patients seeking a more permanent solution to their allergies, as immunotherapy can modify the immune system's response to allergens.  3. **Specific Allergens:** It's most effective for allergies to pollen, dust mites, pet dander, certain molds, and stinging insects.  4. **Avoidance of Long-term Medication Use:** It can be an alternative for people who wish to avoid long-term use of medications.  5. **Severe Allergic Reactions:** Particularly in cases of severe reactions to insect stings, allergen immunotherapy can be life-saving.  6. **Childhood Allergies:** It can be particularly beneficial in children to prevent the progression of allergic diseases, such as the progression from allergic rhinitis to asthma.  7. **Patient Preference:** Some patients prefer the approach of addressing the underlying cause of their allergies rather than ongoing symptom management.  It's important to consult with an allergist or immunologist to determine if allergen immunotherapy is appropriate, as it requires a long-term commitment to treatment and is not suitable for everyone. The decision to start immunotherapy should be based on a detailed medical history, allergy testing results, and patient preference. | The information provided about allergen immunotherapy is based on general medical knowledge and guidelines, commonly accepted practices in the field of allergy and immunology, and the principles taught in medical education. Specifically, these guidelines and practices are in line with the standards set by organizations like the American Academy of Allergy, Asthma & Immunology (AAAAI) and the European Academy of Allergy and Clinical Immunology (EAACI). However, the response does not directly cite specific documents or articles from these organizations. For the most detailed and up-to-date information, it's recommended to refer to the latest guidelines and research articles published by these and similar professional bodies in the field of allergy and immunology. |
| Indications and Contraindications | What are the contraindications of allergen immunotherapy? | Allergen immunotherapy, also known as allergy shots, is a form of long-term treatment that decreases symptoms for many people with allergic rhinitis, allergic asthma, conjunctivitis (eye allergy), or stinging insect allergy. However, it's not suitable for everyone. Here are some of the contraindications:  1. **Severe or Uncontrolled Asthma:** Patients with severe or poorly controlled asthma are at higher risk of serious reactions from allergen immunotherapy.  2. **Heart Diseases:** Certain cardiovascular diseases, especially those requiring beta-blocker medications, may increase the risk of severe systemic reactions.  3. **Beta-Blocker Medications:** These medications can interfere with the treatment of severe allergic reactions and are considered a contraindication.  4. **Autoimmune Diseases:** Conditions such as rheumatoid arthritis or systemic lupus erythematosus might be exacerbated by allergen immunotherapy.  5. **Severe Immunodeficiency:** Individuals with significant immune system deficiencies might not respond adequately to the treatment and are at increased risk of adverse reactions.  6. **Malignancies:** Certain cancers, especially those related to the immune system like lymphoma, may be a contraindication.  7. **Age Factors:** Very young children or elderly patients might have increased risks or decreased benefits from the therapy.  8. **Unwillingness or Inability to Adhere to Treatment Protocol:** Regular and long-term treatment is necessary for effectiveness, so commitment is essential.  9. **Pregnancy:** Generally, it's not recommended to start allergen immunotherapy during pregnancy due to potential risks, but continuation of a treatment already started before pregnancy might be considered.  10. **Severe Allergic Reactions to Previous Immunotherapy:** A history of severe reactions to allergen immunotherapy itself is a contraindication.  Remember, the decision to proceed with allergen immunotherapy should be made after a thorough evaluation by a healthcare professional specialized in allergies, who can assess the benefits and risks based on an individual's medical history and current health status. | The information provided about the contraindications of allergen immunotherapy is based on general medical knowledge and guidelines in the field of allergy and immunology. Specific references include:  1. **Clinical Practice Guidelines:** These are guidelines provided by professional medical associations such as the American Academy of Allergy, Asthma & Immunology (AAAAI) or the European Academy of Allergy and Clinical Immunology (EAACI). They offer comprehensive insights into the best practices for allergen immunotherapy.  2. **Medical Textbooks:** Standard textbooks in the field of allergy and immunology often contain detailed chapters on allergen immunotherapy, including its indications, contraindications, and management strategies.  3. **Peer-Reviewed Journal Articles:** Journals like the "Journal of Allergy and Clinical Immunology" and others frequently publish research studies, reviews, and expert opinions on various aspects of allergen immunotherapy.  4. **Official Health Websites:** Websites like the Mayo Clinic, WebMD, and others provide patient-friendly information that is often based on current medical guidelines and research.  For the most accurate and personalized information, it's always recommended to consult directly with healthcare professionals or specific medical texts or guidelines. |
| Indications and Contraindications | In what patients is it recommended to use sublingual immunotherapy (SLIT)? | Sublingual Immunotherapy (SLIT) is a form of allergy treatment where the patient takes small doses of an allergen under the tongue to boost tolerance to the substance and reduce symptoms. The recommended duration and starting time for SLIT can vary based on individual needs and the specific allergens involved. However, here are some general guidelines:  1. **Duration of Treatment**: The typical duration for SLIT is usually around 3 to 5 years. This is because it takes time for the immune system to build up a tolerance to the allergen. The full benefits of SLIT are often seen after a consistent course of treatment over several years.  2. **Starting Time**: SLIT can be started at any time of the year. However, some doctors might recommend starting the treatment a few months before the allergy season for seasonal allergens (like pollen) to give the immune system time to build up tolerance before the peak allergy season. For perennial allergens (like dust mites), the starting time may be less critical.  It's important to follow the advice of an allergist or healthcare provider who can tailor the treatment plan based on the individual's specific allergy profile and health condition. They can provide guidance on the optimal duration and starting time for SLIT based on the patient's needs. | The information provided about Sublingual Immunotherapy (SLIT) is based on general medical knowledge and guidelines commonly accepted in the field of allergy treatment. I haven't referenced specific external sources for this response, as it reflects standard practices and consensus in the medical community regarding SLIT. However, for the most accurate and personalized information, it's always best to consult directly with a healthcare professional or an allergist. They can offer advice tailored to individual health needs and the specific allergen in question. If you need detailed studies or articles on this topic, I can assist in finding those for you. |
| Indications and Contraindications | What type of immunotherapy  and duration of treatment is recommended for grass pollen-driven allergic rhinitis in adults? | For adults with allergic rhinitis driven by grass pollen, the recommended type of immunotherapy can be either sublingual immunotherapy (SLIT) or subcutaneous immunotherapy (SCIT). Here's a brief overview of each:  1. **Sublingual Immunotherapy (SLIT)**: This involves placing a tablet containing grass pollen extract under the tongue daily. SLIT is often preferred due to its convenience and safety, as it can be administered at home after the first dose, which is typically given under medical supervision. The treatment usually starts before the grass pollen season and continues throughout the season, and may be carried on for three or more years.  2. **Subcutaneous Immunotherapy (SCIT)**: This is the traditional form of allergy shots, where small doses of the allergen are injected under the skin. SCIT generally requires regular visits to a healthcare provider for injections, which are given once or twice a week for about three to six months (the build-up phase), followed by a maintenance phase with less frequent injections (typically every 4-6 weeks) for three to five years.  The duration of both SLIT and SCIT is usually at least three years, as longer treatment has been shown to provide more lasting benefits and may lead to long-term remission of allergy symptoms.  It's important to note that the specific treatment plan, including the type of immunotherapy and duration, should be personalized based on the individual's medical history, severity of symptoms, and response to therapy. A healthcare provider or an allergist can provide the most appropriate recommendation based on these factors. | I should clarify that the information provided about immunotherapy for grass pollen-driven allergic rhinitis in adults is based on general medical knowledge and guidelines in the field of allergy and immunotherapy, up to my last training data in April 2023. It includes widely accepted practices and treatments such as sublingual immunotherapy (SLIT) and subcutaneous immunotherapy (SCIT), and the typical durations for these treatments. However, for the most current and personalized medical advice, it's always recommended to consult directly with healthcare professionals. They can provide the latest information and tailor treatments to individual needs and medical histories. |
| Indications and Contraindications | What type of immunotherapy and duration of treatment is recommended for perennial house dust mite allergic rhinitis in adults? | For the treatment of perennial house dust mite allergic rhinitis in adults, immunotherapy can be an effective option. There are two main types of immunotherapy used in this context:  1. **Subcutaneous Immunotherapy (SCIT):** This traditional form involves regular injections of allergen extracts under the skin. The initial phase, known as the build-up phase, typically involves injections 1-2 times per week for about 3-6 months. Once the effective dose is reached, the maintenance phase begins, where injections are less frequent, usually once a month. This phase generally continues for 3-5 years.  2. **Sublingual Immunotherapy (SLIT):** This is a newer form of immunotherapy where allergen extracts are taken orally, usually in the form of drops or tablets placed under the tongue. The initial doses might be daily for the first few days, followed by a long-term maintenance phase which involves daily treatment. The duration of SLIT is usually around 3-5 years, similar to SCIT.  The duration of treatment can vary based on individual patient responses and the severity of symptoms. Generally, a minimum of three years is recommended to achieve long-term benefits and potential lasting remission of allergy symptoms.  It's important to note that the effectiveness of immunotherapy can vary from person to person, and it's crucial to undergo these treatments under the supervision of a healthcare professional specialized in allergies. Regular follow-ups are important to monitor response to treatment and adjust doses as necessary. Additionally, not every patient is a suitable candidate for immunotherapy, and decisions should be made based on a thorough evaluation of the individual's medical history, allergy profile, and overall health status. | In providing information about the immunotherapy options for perennial house dust mite allergic rhinitis in adults, the response draws on widely accepted medical guidelines and practices in the field of allergy and immunotherapy. These include:  1. **Clinical Guidelines and Research in Allergy and Immunology:** The recommendations for subcutaneous immunotherapy (SCIT) and sublingual immunotherapy (SLIT) are based on standard practices as outlined in clinical guidelines and research studies in the field of allergy and immunology. These guidelines are regularly updated by professional organizations such as the American Academy of Allergy, Asthma & Immunology (AAAAI) and the European Academy of Allergy and Clinical Immunology (EAACI).  2. **Textbooks and Medical Literature:** The duration and methodology of SCIT and SLIT treatments are standard medical knowledge, commonly referenced in medical textbooks and literature related to allergy treatment.  3. **Expert Consensus:** The recommendations also reflect the consensus among healthcare professionals specializing in allergy and immunology, drawing from their clinical experience and the outcomes observed in patients undergoing immunotherapy for house dust mite allergies.  Please note that while this information reflects the general practices and guidelines in the field, specific treatment plans should always be personalized based on individual patient needs and under the guidance of a healthcare professional. |
| Standardization and Efficacy | What is the importance of using standardized allergen products for allergen immunotherapy? | The use of standardized allergen products in allergen immunotherapy (AIT) is crucial for several reasons:  1. **Accuracy and Consistency in Dosage**: Standardized products ensure that each dose contains a precise and consistent amount of the allergen. This is vital for gradually increasing the dose to build tolerance in the patient's immune system without triggering severe allergic reactions.  2. **Safety**: Standardization reduces the risk of adverse reactions. Non-standardized extracts can have variable allergen content, increasing the risk of administering too much or too little of the allergen, potentially leading to ineffective treatment or severe allergic reactions.  3. **Efficacy**: Standardized allergens provide a reliable measure of effectiveness. With consistent allergen levels, the effectiveness of AIT can be more accurately assessed and compared across different studies and clinical practices.  4. **Quality Control**: Standardization involves rigorous quality control measures, ensuring that the allergen extracts are free from contaminants and have a consistent potency, which is crucial for patient safety and treatment efficacy.  5. **Regulatory Compliance**: Standardized products are often required by regulatory agencies. This compliance ensures that the products meet specific safety and efficacy standards, providing an additional layer of protection for patients.  6. **Comparability and Research**: Using standardized products allows for more meaningful comparisons in clinical trials and research studies. This can lead to a better understanding of AIT, improvements in treatment protocols, and the development of new therapies.  7. **Patient Confidence**: Knowing that they are receiving a standardized and regulated product can increase patient confidence in the safety and efficacy of their treatment.  Overall, the use of standardized allergen products in AIT enhances the treatment's safety, efficacy, and reliability, contributing significantly to the overall success and advancement of allergen immunotherapy. | The information provided about the importance of using standardized allergen products for allergen immunotherapy doesn't come from specific references, but rather is based on general principles of medical treatment, pharmacology, and immunotherapy. This knowledge is derived from established practices and understandings in the fields of allergy and immunotherapy, which emphasize the need for precision, safety, and efficacy in treatments. For the most accurate and detailed information, it's advisable to consult specific medical texts, peer-reviewed journals, or guidelines from professional medical organizations specializing in allergies and immunotherapy. |
| Standardization and Efficacy | Which are effective pollen mixtures frequently used for allergen immunotherapy? | Allergen immunotherapy, commonly known as allergy shots, involves exposing patients to increasing amounts of allergens to build up their tolerance. The effective pollen mixtures used in this therapy typically depend on the specific allergies of the patient and the prevalent allergens in the region. However, some commonly used pollen mixtures include:  1. **Grass Pollen Mixtures**: These are among the most common, especially for people allergic to grass pollens like Timothy, Bermuda, or Rye.  2. **Tree Pollen Mixtures**: For those allergic to tree pollens, mixtures might include common culprits like Birch, Oak, Alder, and Cedar.  3. **Weed Pollen Mixtures**: Weeds such as Ragweed, Mugwort, and Nettle are often included in immunotherapy for patients with these specific allergies.  4. **Mixed Pollen Extracts**: In some cases, extracts from different groups (grass, tree, and weed) are combined, especially if the patient is allergic to multiple types of pollen.  5. **Region-Specific Mixtures**: Based on geographical location and local flora, custom mixtures are created to target the most prevalent pollens in that area.  It's important to note that the effectiveness of these mixtures can vary, and they should be administered under the guidance of an allergy specialist who can tailor the therapy to the patient's specific needs. The composition of these mixtures might also change over time as new research emerges and as the prevalence of different types of pollen changes. | The information provided about effective pollen mixtures for allergen immunotherapy is based on common medical knowledge and standard practices in allergy treatment. It does not come from specific references but rather from a general understanding of allergology and immunotherapy principles. For the most accurate and personalized information, consulting with an allergy specialist or reviewing current medical literature on allergen immunotherapy would be advisable. |
| Standardization and Efficacy | Does the efficacy of allergen immunotherapy change if a patient with allergic rhinitis has also co-existing asthma? | Allergen immunotherapy, commonly used for treating allergic rhinitis, can also have implications for patients with co-existing asthma. The efficacy of this therapy in such cases can indeed vary. Here's a general overview:  1. **Enhanced Efficacy**: For some patients, allergen immunotherapy for allergic rhinitis can also alleviate asthma symptoms. This is particularly true if the asthma is primarily triggered by the same allergens causing the rhinitis. The immunotherapy can reduce the overall allergic response, benefiting both conditions.  2. **Complex Interactions**: The presence of asthma can sometimes make allergic rhinitis more severe, and vice versa. This interplay can affect how well immunotherapy works. It's important for the treatment plan to consider both conditions.  3. **Safety Concerns**: In patients with severe or uncontrolled asthma, allergen immunotherapy must be administered with caution. There's a risk of triggering asthma attacks, especially during the initial phase of the treatment. This requires careful monitoring and adjustment of the therapy by healthcare professionals.  4. **Customized Approach**: The treatment plan for a patient with both allergic rhinitis and asthma may need to be more personalized. The type, dose, and duration of allergen immunotherapy might be adjusted based on the patient's response and the severity of both conditions.  5. **Potential for Long-Term Benefits**: Studies suggest that successful allergen immunotherapy in allergic rhinitis patients can potentially prevent the development of asthma or reduce its severity in the long term. This is especially significant for children and young adults.  6. **Interdisciplinary Care**: Managing patients with both conditions often requires a collaborative approach, involving allergists, pulmonologists, and primary care providers.  7. **Ongoing Research**: The field is continually evolving, with research focused on understanding the best practices for treating patients with co-existing allergic rhinitis and asthma.  Each patient's response to allergen immunotherapy can be quite individual, and co-existing asthma adds an additional layer of complexity. It's crucial for patients to be under the care of healthcare professionals who can closely monitor and adjust treatment as needed. | The information I provided about the efficacy of allergen immunotherapy in patients with allergic rhinitis and co-existing asthma is based on general medical knowledge and principles in the fields of allergology and pulmonology. These insights are derived from a combination of established medical guidelines, research studies, and clinical practices in these areas. However, I did not refer to specific references or recent studies in my response. For the most current and detailed information, consulting recent medical literature, clinical guidelines, or consulting with healthcare professionals specializing in allergy and asthma would be recommended. |
| Special Populations | Can allergen immunotherapy be given to pediatric patients with allergic rhinitis? | Yes, allergen immunotherapy can be given to pediatric patients with allergic rhinitis. This treatment, also known as allergy shots or desensitization therapy, is often used when other treatments like medications do not control symptoms adequately, or if medication side effects are problematic.  The therapy involves regularly administering gradually increasing amounts of the allergen, with the aim to desensitize the immune system. It can be highly effective in reducing allergy symptoms and in some cases, it can lead to long-lasting relief even after treatment has ended.  However, it's important to note that allergen immunotherapy must be administered under the guidance of a healthcare professional experienced in allergy treatment, as there are risks of allergic reactions, including anaphylaxis. The decision to start allergen immunotherapy in pediatric patients depends on several factors, including the age of the child, the severity of symptoms, the specific allergen(s), and the child's ability to adhere to the treatment protocol. | The information about allergen immunotherapy for pediatric patients with allergic rhinitis is based on general medical knowledge and guidelines in the field of allergy and immunology. Specific references include:  1. **American Academy of Allergy, Asthma & Immunology (AAAAI)**: AAAAI provides comprehensive guidelines and position statements regarding the use of allergen immunotherapy, including its application in pediatric patients.  2. **Journal Articles and Studies in Allergy and Clinical Immunology**: Peer-reviewed medical journals often publish studies and reviews about the effectiveness and safety of allergen immunotherapy in children.  3. **Clinical Guidelines from the American College of Allergy, Asthma & Immunology (ACAAI)**: The ACAAI offers detailed clinical guidelines and practice parameters for allergen immunotherapy, including considerations for pediatric patients.  4. **Pediatric Allergy and Immunology Textbooks**: Medical textbooks specifically focused on pediatric allergy and immunology cover the use of allergen immunotherapy in children in detail, outlining protocols, safety considerations, and efficacy.  5. **European Academy of Allergy and Clinical Immunology (EAACI)**: The EAACI also provides guidelines and position papers on allergen immunotherapy, including its use in children.  These sources collectively represent the consensus and practices in the field of allergy and immunology, particularly regarding the treatment of allergic rhinitis in pediatric patients. |
| Special Populations | Is the use of allergen immunotherapy recommended in otherwise healthy elderly adults with allergic rhinitis? | Allergen immunotherapy (AIT) is considered an important, guideline-approved therapy for allergic rhinitis (AR) and asthma, and it can modify the disease’s natural history with long-lasting effects on symptoms after the treatment has ended. While traditionally, AIT has been mostly reserved for younger populations, recent studies and evaluations have shown its efficacy and safety in elderly patients as well.  A notable study conducted by Bozek et al. focused on elderly patients (aged 60 years and older) with allergic rhinitis due to house dust mite allergens. This study, which was a 3-year double-blind, placebo-controlled trial, found that sublingual immunotherapy (SLIT) with house dust mite extracts resulted in significant clinical improvement in the active group compared to the placebo group. The treatment was well-tolerated, leading to a reduction in nose-related symptoms and medication scores, particularly during periods of the year when heating was on.  Furthermore, the GA^2LEN/EAACI pocket guide on AIT for AR and asthma indicates that there is no upper age limit for prescribing AIT. The only absolute contraindications for AIT are the presence of uncontrolled asthma, autoimmune disorders not responding to therapy, active immune-deficiency, and active malignancies. These conditions are more frequent among elderly individuals, so a detailed medical history is essential before prescribing AIT. Additionally, cardiovascular diseases such as unstable angina, recent myocardial infarction, significant arrhythmia, and uncontrolled systemic hypertension are considered contraindications.  In summary, while specific guidelines for AIT in elderly patients are not yet fully established, the existing evidence suggests that it can be an effective and safe treatment option for otherwise healthy elderly adults with allergic rhinitis, provided there are no contraindications based on individual health conditions. | The information provided is based on the following references:  1. An article from Clinical and Molecular Allergy discussing allergen immunotherapy in the elderly and its implications on immune system changes commonly seen in older adults https://clinicalmolecularallergy.biomedcentral.com/articles/10.1186/s12948-017-0075-2.  2. A study published in Clinical and Experimental Allergy, which evaluated the clinical efficacy of sublingual immunotherapy with house dust mite extracts in elderly patients with allergic rhinitis. This study demonstrated significant clinical improvement in the group receiving active treatment compared to a placebo group. https://pubmed.ncbi.nlm.nih.gov/23829621/ |
| Special Populations | Is it recommended to initiate allergen immunotherapy regimen for allergic rhinitis during pregnancy? | It's generally not recommended to initiate allergen immunotherapy (also known as allergy shots) for allergic rhinitis during pregnancy. This is primarily due to the lack of substantial research on the safety and efficacy of starting this treatment during pregnancy. Here are a few considerations:  1. **Potential Risks**: The primary concern with starting allergen immunotherapy during pregnancy is the risk of anaphylaxis, a severe allergic reaction. Anaphylaxis is rare but can be more complicated to manage during pregnancy.  2. **Limited Research**: There's limited research on the effects of starting allergen immunotherapy during pregnancy. Most studies and clinical guidelines focus on women who were already receiving the therapy before becoming pregnant.  3. **Continuation vs. Initiation**: While initiating allergen immunotherapy during pregnancy is usually not recommended, continuing a pre-existing regimen may be considered safe. This is because the body is already accustomed to the allergens being introduced, and the doses are typically in a maintenance phase rather than the initial build-up phase.  4. **Consultation with Healthcare Providers**: It's important for pregnant women to consult with their allergist and obstetrician before making decisions about allergen immunotherapy. The healthcare providers will consider the severity of the allergic symptoms, the benefits of treatment, and the potential risks to both the mother and the fetus.  5. **Alternative Treatments**: For managing allergic rhinitis during pregnancy, healthcare providers might recommend safer alternatives, like nasal saline irrigation or certain antihistamines that are considered safe during pregnancy.  It's crucial for pregnant women to discuss all treatment options with their healthcare providers to ensure the safety of both the mother and the developing fetus. | The information provided about the safety and considerations for initiating allergen immunotherapy during pregnancy is based on general medical guidelines and practices commonly accepted in the field of allergy and immunology. These guidelines are informed by a body of clinical research and expert consensus. Specific sources for this information include:  1. **Clinical Immunology Textbooks**: These textbooks often provide detailed chapters on allergen immunotherapy, including special considerations for pregnant patients.  2. **Medical Guidelines from Allergy and Immunology Societies**: Organizations like the American Academy of Allergy, Asthma & Immunology (AAAAI) and the European Academy of Allergy and Clinical Immunology (EAACI) publish guidelines that discuss the management of allergic diseases during pregnancy.  3. **Peer-Reviewed Medical Research**: Studies published in medical journals that have explored the safety and efficacy of allergen immunotherapy during pregnancy.  4. **Practice Experience**: The standard practices and recommendations in allergology, as observed in clinical settings.  Please note, while these references inform the general guidance provided, for personalized medical advice, it's always best to consult directly with healthcare professionals. |
| Indications and Contraindications | What is the recommended duration of allergen immunotherapy in order to achieve long-term efficacy after discontinuation in patients with allergic rhinitis? | The recommended duration of allergen immunotherapy (AIT) for achieving long-term efficacy after discontinuation in patients with allergic rhinitis is typically around 3 to 5 years. This recommendation is based on various factors:  1. **Clinical Studies**: Research has shown that a minimum of three years of continuous allergen immunotherapy is often necessary to induce long-term clinical remission and immunologic changes that can last even after the treatment is stopped.  2. **Guidelines from Professional Organizations**: Bodies like the American Academy of Allergy, Asthma & Immunology (AAAAI) and the European Academy of Allergy and Clinical Immunology (EAACI) often provide guidelines based on the latest research. These guidelines generally recommend a minimum of 3 years of treatment.  3. **Individual Response**: The exact duration can vary depending on the individual's response to the therapy. Some patients may need longer treatment for optimal results.  4. **Type of Allergen**: The duration might also vary depending on the specific allergen. For example, treatment for certain allergens like bee venom might differ in duration from treatment for environmental allergens.  5. **Maintenance Phase Achieved**: Before discontinuing, it is important that the patient has reached and maintained the maximum tolerated dose for a period of time, as this is considered to be a key factor in achieving long-term efficacy.  It's important to note that the decision to stop treatment should be individualized and made in consultation with a healthcare provider, based on the patient's symptoms, response to therapy, and preference. | The information about the recommended duration of allergen immunotherapy for allergic rhinitis is derived from established medical guidelines and research in the field of allergy and immunology. The key sources for this information include:  1. **Clinical Practice Guidelines**: Guidelines issued by professional bodies like the American Academy of Allergy, Asthma & Immunology (AAAAI) and the European Academy of Allergy and Clinical Immunology (EAACI). These guidelines are based on a comprehensive review of existing research and clinical trials.  2. **Scientific Research and Studies**: Published studies in peer-reviewed medical journals that have investigated the efficacy and optimal duration of allergen immunotherapy.  3. **Immunology and Allergy Textbooks**: Standard medical textbooks that provide detailed information on the practice of allergen immunotherapy, including duration and efficacy.  4. **Expert Consensus**: Recommendations made by experts in the field, often based on a combination of research data, clinical trials, and clinical practice experience.  It's important to remember that specific treatment plans, including the duration of allergen immunotherapy, should always be personalized based on the individual patient's needs and circumstances, in consultation with healthcare professionals. |
| Indications and Contraindications | Is controlled asthma a contraindication to receiving allergen immunotherapy? | Controlled asthma is generally not considered a contraindication to receiving allergen immunotherapy (AIT). In fact, allergen immunotherapy can be beneficial for individuals with asthma, as it may help in reducing asthma symptoms and the need for medications by desensitizing the individual to specific allergens.  However, it's important to note that there are certain conditions where allergen immunotherapy should be administered with caution or may be contraindicated:  1. **Uncontrolled or Severe Asthma**: Patients with uncontrolled or severe asthma are at a higher risk of experiencing severe allergic reactions, including anaphylaxis, during allergen immunotherapy. Therefore, it's crucial that asthma is well-controlled before starting AIT.  2. **Special Considerations**: Other factors, such as the presence of coexisting medical conditions, the severity of allergic reactions, and the patient’s ability to adhere to treatment protocols, also play a role in determining the suitability of AIT.  3. **Monitoring**: Close monitoring, especially during the initial phase of treatment, is important. Dosage adjustments may be necessary based on the patient's response to the therapy.  It is essential for individuals considering allergen immunotherapy to discuss their specific situation with their healthcare provider. The decision to proceed with AIT should be made after a thorough evaluation of the individual's medical history, current health status, and the potential risks and benefits of the therapy. | The information provided about allergen immunotherapy and its relationship to asthma is based on established medical knowledge and guidelines. Here are some of the key references and sources that inform these guidelines and best practices:  1. **Global Initiative for Asthma (GINA)**: GINA provides global strategies for asthma management and prevention, which include recommendations on the use of allergen immunotherapy in asthma patients.  2. **American Academy of Allergy, Asthma & Immunology (AAAAI)**: AAAAI offers extensive resources and guidelines on the management of asthma and the use of allergen immunotherapy.  3. **European Academy of Allergy and Clinical Immunology (EAACI)**: EAACI provides guidelines and position papers on various aspects of allergy treatments, including immunotherapy.  4. **Clinical research studies and medical journals**: Various studies published in reputable medical journals provide insights into the efficacy and safety of allergen immunotherapy in asthma patients.  5. **Expert consensus reports and reviews**: Consensus reports and reviews by experts in the field summarize the current understanding and recommendations for the use of allergen immunotherapy.  These sources are foundational in the field of allergy and asthma treatment and are regularly updated to reflect the latest research and clinical practices. |
| Safety and Adverse Reactions | What are the most common adverse reactions to subcutaneous immunotherapy? | Subcutaneous immunotherapy, commonly known as allergy shots, typically involves a series of injections containing tiny amounts of the allergens to which a person is allergic. The most common adverse reactions to this treatment can be categorized into local and systemic reactions:  1. **Local Reactions:** These are the most common and usually occur at the site of the injection. They can include:  - Redness  - Swelling  - Itching  - Pain or discomfort   Local reactions are generally mild and often resolve within a few hours to a few days.  2. **Systemic Reactions:** These are less common but more serious and can affect the whole body. They include:  - Hives or rash  - Wheezing or shortness of breath  - Nasal congestion  - Sneezing  - Itchy or watery eyes  - Anaphylaxis (a rare but severe allergic reaction that can be life-threatening)   Systemic reactions typically require immediate medical attention.  It's important for patients receiving subcutaneous immunotherapy to be monitored for a period after each injection due to the potential for systemic reactions. Additionally, the dosing and schedule of the injections are carefully tailored to each individual to minimize the risk of adverse reactions while maximizing the treatment's effectiveness. | The information I provided about the common adverse reactions to subcutaneous immunotherapy is based on general medical knowledge and standard practices in allergy treatment. This information is commonly found in medical textbooks, allergy treatment guidelines, and health information websites. Specific references include:  1. "Middleton's Allergy: Principles and Practice" - a comprehensive textbook on allergy and immunology. 2. Clinical guidelines from professional organizations such as the American Academy of Allergy, Asthma & Immunology (AAAAI) or the European Academy of Allergy and Clinical Immunology (EAACI). 3. Health information websites like Mayo Clinic, WebMD, or Healthline, which provide patient-friendly explanations of medical treatments and their side effects.  These sources are widely recognized for their reliability and authority in the field of allergy and immunology. |
| Safety and Adverse Reactions | At what time can most adverse reactions occur after the application of subcutaneous immunotherapy? | Most adverse reactions to subcutaneous immunotherapy (SCIT) typically occur within the first 30 minutes after the injection. This is why it is generally recommended that patients remain in the clinic for at least 30 minutes post-injection so they can be monitored for any immediate reactions. However, it's important to note that reactions can still occur after this period, albeit less frequently. Delayed reactions can happen hours or even days after the treatment, though these are rarer. As always, patients should be informed about the signs of adverse reactions and advised to seek medical attention if they experience any concerning symptoms. | The information provided about the timing of adverse reactions to subcutaneous immunotherapy (SCIT) is based on established medical guidelines and common practices in allergy treatment. These guidelines are widely referenced in medical literature and are a part of standard training for healthcare professionals involved in administering SCIT. Specific references include:  1. **Clinical Guidelines and Practice Parameters**: Various medical organizations, such as the American Academy of Allergy, Asthma & Immunology (AAAAI) and the European Academy of Allergy and Clinical Immunology (EAACI), provide guidelines on SCIT administration, including safety protocols.  2. **Medical Textbooks and Journals**: Standard immunology and allergy textbooks, as well as peer-reviewed medical journals, often discuss the protocols and safety measures associated with SCIT, including the timing and management of adverse reactions.  3. **Patient Education Resources**: Hospitals and allergy clinics provide educational materials to patients undergoing SCIT, which typically include information about the risk of adverse reactions and the recommended observation period after injections.  While these references form the basis of the provided information, if you need specific studies or documents, I can conduct an online search for more detailed sources. |
| Safety and Adverse Reactions | Which premedication can be used to reduce the severity of local and systemic cutaneous reactions to allergen immunotherapy? | Premedication to reduce the severity of local and systemic cutaneous reactions to allergen immunotherapy typically includes antihistamines. Non-sedating antihistamines like cetirizine, loratadine, or fexofenadine are often recommended because they can help mitigate allergic reactions without causing significant drowsiness. In some cases, physicians might also recommend leukotriene receptor antagonists like montelukast.  These premedications are generally taken regularly during the course of allergen immunotherapy. They work by blocking the effects of histamine, a substance in the body that is released during allergic reactions, thereby reducing symptoms such as itching, swelling, and rashes.  However, the exact premedication regimen can vary based on individual patient needs, the type of allergen immunotherapy being used, and the severity of the allergic reactions. It's important for patients to discuss with their healthcare provider the best premedication strategy for their specific situation. | The information I provided about premedication for allergen immunotherapy is based on general medical knowledge and guidelines in the field of allergy and immunotherapy. This knowledge comes from a variety of medical sources, including allergy treatment guidelines, pharmacology textbooks, and clinical studies on the efficacy of antihistamines and leukotriene receptor antagonists in allergen immunotherapy. Specific references include:  1. "Middleton's Allergy: Principles and Practice" – a comprehensive textbook on allergy and immunology that details various treatment strategies, including premedication for allergen immunotherapy. 2. Clinical guidelines from professional bodies such as the American Academy of Allergy, Asthma & Immunology (AAAAI) or the European Academy of Allergy and Clinical Immunology (EAACI), which provide recommendations for allergen immunotherapy management. 3. Pharmacological textbooks and resources like "Goodman & Gilman's The Pharmacological Basis of Therapeutics" which describe the mechanism of action and uses of antihistamines and leukotriene receptor antagonists.  These sources collectively contribute to the understanding of the best practices in premedication for allergen immunotherapy. |
| Safety and Adverse Reactions | Should premedication with an oral H1 antihistamine be recommended before allergen immunotherapy? | Premedication with an oral H1 antihistamine before allergen immunotherapy (AIT) is often considered as a strategy to minimize allergic reactions, which can occur as a side effect of AIT. The effectiveness and recommendation of premedication can depend on several factors:  1. **Type of Allergen Immunotherapy**: The necessity and benefits of premedication might vary between subcutaneous immunotherapy (SCIT) and sublingual immunotherapy (SLIT).  2. **Patient's Allergy History**: Patients with a history of severe allergic reactions may be more likely to benefit from premedication.  3. **Severity and Type of Allergic Symptoms**: The presence of significant allergic rhinitis or asthma symptoms can influence the decision.  4. **Patient's Tolerance to Antihistamines**: Some patients may experience side effects from antihistamines, which could affect their suitability as premedication.  5. **Guidelines and Protocols**: Different health organizations or allergy societies might have varying guidelines regarding premedication.  6. **Clinical Evidence**: The decision should be informed by the latest research and clinical trials that explore the efficacy and safety of premedication in AIT.  Consultation with a healthcare professional, preferably an allergist or immunologist, is essential for personalized advice. They can assess the individual's specific situation and consider the latest clinical guidelines and research to make a recommendation. | The reference I used for the information provided is:  - PubMed: This reference discusses the enhancement of the efficacy of specific-allergen immunotherapy through premedication with antihistamines, specifically in the context of Hymenoptera venom allergy https://pubmed.ncbi.nlm.nih.gov/11149995/ |
| Standardization and Efficacy | What are the preventive effects of allergen immunotherapy for children and adolescents with allergic rhinitis driven by pollen allergy? | Allergen immunotherapy, also known as allergy shots, for children and adolescents with allergic rhinitis caused by pollen allergies can have several preventive effects:  1. **Long-term Relief**: Immunotherapy can lead to a significant reduction in symptoms over the long term. By gradually introducing the body to the allergen, it helps to build tolerance and reduce the sensitivity to the pollen.  2. **Reduction in Medication Use**: Many patients find that they need less allergy medication to control their symptoms after undergoing immunotherapy.  3. **Prevention of Asthma Development**: There is evidence that allergen immunotherapy in children with allergic rhinitis may prevent the development of asthma, which is often associated with untreated allergies.  4. **Improved Quality of Life**: By reducing symptoms, immunotherapy can significantly improve the overall quality of life, including better sleep and fewer limitations on outdoor activities.  5. **Prevention of New Sensitizations**: Immunotherapy may help in preventing the development of new allergies in some children and adolescents.  6. **Long-lasting Effects**: The beneficial effects of allergen immunotherapy can last for years, even after the treatment has ended, providing long-term relief from allergy symptoms.  It's important to note that the effectiveness and outcomes of immunotherapy can vary among individuals. A healthcare professional can provide personalized advice based on the specific needs and medical history of the child or adolescent. | The information I provided about the preventive effects of allergen immunotherapy for children and adolescents with allergic rhinitis due to pollen allergy is based on established medical knowledge and guidelines up to my last training data in April 2023. These insights are derived from a general understanding of allergen immunotherapy's mechanisms and outcomes as documented in medical literature, including:  - Clinical guidelines and research studies published in medical journals such as the Journal of Allergy and Clinical Immunology. - Health information from reputable medical sources like the Mayo Clinic, WebMD, and the American Academy of Allergy, Asthma & Immunology. - Textbooks and articles on immunotherapy and allergic diseases.  As an AI developed by OpenAI, I don't have the capability to browse the internet or access external databases, so I can't provide specific article titles or authors. For the most current and detailed information, consulting recent medical literature or a healthcare professional is recommended. |
| Safety and Adverse Reactions | Which are risk factors for the appearance of systemic reactions during allergen immunotherapy? | Allergen immunotherapy, also known as allergy shots, can be highly effective for certain types of allergies, but like any medical treatment, it carries some risks of adverse reactions, including systemic reactions. The risk factors for systemic reactions during allergen immunotherapy include:  1. **High Allergen Sensitivity:** Patients with a high degree of sensitivity to the allergen being treated are at an increased risk.  2. **Uncontrolled Asthma:** Individuals with asthma that is not well-controlled are more prone to systemic reactions. Asthma control should be assessed and optimized before starting immunotherapy.  3. **Dosage Errors:** Incorrect dosing, either due to a mistake or a misunderstanding of the proper dosage, can increase the risk.  4. **Concurrent Illnesses:** Having a respiratory infection or fever at the time of treatment may increase the risk of a systemic reaction.  5. **Rapid Increase in Allergen Dose:** Escalating the allergen dose too quickly can increase the risk of a systemic reaction. A gradual increase is usually safer.  6. **Inadequate Waiting Period After Injection:** It's generally recommended to wait in the healthcare provider's office for a period after the injection, as most serious reactions occur shortly after administration.  7. **Exercise Around the Time of Injection:** Vigorous physical activity either before or after the allergen injection can increase the risk of systemic reactions.  8. **Injection Technique Errors:** Incorrect injection techniques can contribute to increased risk.  9. **Premenstrual Phase in Females:** Some studies suggest that the premenstrual phase may be associated with an increased risk of systemic reactions.  10. **Concomitant Medications:** Certain medications, like beta-blockers, may increase the risk of systemic reactions or may interfere with the treatment of these reactions.  It's important for patients to discuss their specific risk factors with their healthcare provider before starting allergen immunotherapy. The provider can take steps to minimize risks, such as adjusting the treatment protocol or addressing controllable risk factors like uncontrolled asthma. | The information provided about the risk factors for systemic reactions during allergen immunotherapy is based on general medical knowledge and guidelines in the field of allergy and immunology. This information is typically covered in medical textbooks, clinical guidelines, and review articles on allergen immunotherapy. Specific sources for this information include:  1. **Clinical Guidelines and Consensus Statements:** Guidelines from professional bodies like the American Academy of Allergy, Asthma & Immunology (AAAAI) or the European Academy of Allergy and Clinical Immunology (EAACI).  2. **Medical Textbooks:** Standard textbooks in the field of allergy and immunology often discuss allergen immunotherapy and its associated risks.  3. **Peer-Reviewed Medical Journals:** Research articles and reviews in journals such as the "Journal of Allergy and Clinical Immunology" or "Annals of Allergy, Asthma & Immunology" provide detailed information on allergen immunotherapy.  4. **Expert Opinions:** Opinions and experiences of healthcare professionals specialized in allergy and immunology.  Since I don't have access to external databases or the internet to retrieve specific articles or documents, the information shared is based on the generalized and consensus understanding in the medical community as of my last training data in April 2023. For the most current and specific references, consulting recent medical literature or guidelines from relevant medical bodies is recommended. |
| Safety and Adverse Reactions | Is allergen immunotherapy considered safe and well-tolerated in patients? | Allergen immunotherapy, also known as allergy shots or desensitization therapy, is generally considered safe and well-tolerated for most patients with certain types of allergies. However, it's important to note that while it is safe for many, it does carry some risks and is not suitable for everyone. Here are some key points to consider:  1. **Effectiveness**: Allergen immunotherapy is effective in reducing symptoms for many types of allergies, such as those to pollen, dust mites, animal dander, and insect stings. It's also used for allergic asthma.  2. **Safety Profile**: The treatment is generally safe when administered under the guidance of a trained allergist. Patients are usually monitored for a short period after receiving the injection to manage any immediate adverse reactions.  3. **Side Effects**: Common side effects are usually mild and may include redness and swelling at the injection site, itching, and sneezing. In rare cases, more severe reactions like anaphylaxis can occur, but this is uncommon, especially with proper monitoring and dosing adjustments.  4. **Not Suitable for Everyone**: Allergen immunotherapy is not recommended for individuals with certain medical conditions, like severe asthma or heart problems. It's also typically not used in very young children.  5. **Long-Term Commitment**: It requires a long-term commitment, often 3-5 years, to achieve lasting benefits. This includes regular injections followed by a maintenance phase.  6. **Individual Response**: The response to allergen immunotherapy can vary widely among individuals. Some may experience significant relief from allergy symptoms, while others may see less benefit.  7. **New Developments**: Sublingual immunotherapy, where allergens are administered under the tongue, is a newer method that's gaining popularity due to its convenience and safety profile, especially for certain allergens like grass pollen.  Before starting any form of allergen immunotherapy, it's crucial to have a thorough evaluation by an allergist to determine if it's an appropriate and safe option for you. They can assess your specific allergies, health history, and other factors to make an informed recommendation. | The information provided is based on my training data, which includes a wide range of medical textbooks, guidelines, and scientific literature available up to April 2023. Specific references include:  1. Medical textbooks and literature that discuss the principles of allergology and immunotherapy. 2. Clinical guidelines and consensus statements from professional bodies such as the American Academy of Allergy, Asthma & Immunology (AAAAI) and the European Academy of Allergy and Clinical Immunology (EAACI). 3. Research studies and reviews published in medical journals that evaluate the efficacy and safety of allergen immunotherapy.  These sources collectively contribute to the understanding of allergen immunotherapy's safety, efficacy, and application in clinical practice. However, for the most current and personalized advice, consulting with a healthcare provider or an allergist is recommended, as they can provide information tailored to individual needs and the latest clinical guidelines. |
